# Potential biases in the classification, analysis and interpretations in cross-sectional study: commentaries – surrounding the article "resting heart rate: its correlations and potential for screening metabolic dysfunctions in adolescents"

**DOI:** 10.1186/1471-2431-14-117

**Published:** 2014-05-03

**Authors:** Augusto César Ferreira de Moraes, Alex Jones Flores Cassenote, Luis A Moreno, Heráclito Barbosa Carvalho

**Affiliations:** 1 Department of Preventive Medicine, School of Medicine of the University of São Paulo, São Paulo, SP, Brazil; 2 GENUD - Growth, Exercise, Nutrition and Development, UNIZAR/Spain, Zaragoza, Spain; 3 Postgraduate Program in Infectious and Parasitic Diseases, School of Medicine of the University of São Paulo, São Paulo, SP, Brazil; 4 Faculty of Health Sciences of the University of Zaragoza, Zaragoza, Spain

**Keywords:** Resting heart rate, Screening test, P-value, High glucose, High triglycerides

## Abstract

**Background:**

Resting heart rate reflects sympathetic nerve activity. A significant association between resting heart rate (HR) and all causes of cardiovascular mortality has been reported by some epidemiologic studies. Despite suggestive evidence, resting heart rate (RHR) has not been formally explored as a prognostic factor and potential therapeutic outcome and, therefore, is not generally accepted in adolescents.

**Discussion:**

The core of the debate is the methodological aspects used in "Resting heart rate: its correlations and potential for screening metabolic dysfunctions in adolescents"; the points are: cutoff used for cluster RHR, two different statistical models used to analyze the same set of variables, one for continuous data, and another for categorical data; interpretation of p-value < 0.05, sampling process involving two random stages, analysis of design effect and the parameters of screening tests.

**Summary:**

Aspects that must be taken into account for evaluation of a screening test to measure the potential for discrimination for a common variable (population with outcome vs. no outcome population), the main indicators are: sensitivity, specificity, accuracy, positive predictive value and negative predictive value. The measures of argumentation equality (CI) or difference (p-valor) are important to validate these indicators but do not indicate quality of screening.

## Background

Recently, Fernandes et al. published an article aimed at analyzing the potential effects of screening and resting heart rate (RHR) on cardiometabolic risk in adolescents [1] in this respected journal. We read the manuscript with great interest, since RHR reflects sympathetic nerve activity [2,3], and it is an easily accessible clinical measurement. A significant association between resting HR and all-causes of cardiovascular mortality has been reported in some epidemiological studies [2,4-6].

After studying the article, we decided to take the opportunity to propose a healthy debate on the methodological aspects used by Fernandes et al. [1]. With this debate, we hope to contribute to the enrichment of the reader, especially with regard to statistical analysis and interpretation of results.

The aim of this article is to present a critical appraisal of methodological aspects of the article "Resting heart rate: its correlations and potential for screening metabolic dysfunctions in adolescents" presented by BMC *Pediatrics*.

## Discussion

First, with regard to the manuscript methodology, what drew our attention was the cutoff used for cluster RHR. We see that the authors used cutoffs developed by the group of the first author (Fernandes RA) [7]. These cutoff points were developed by percentile distribution of a sample composed only of children and adolescent males and the study published in this journal is composed only of adolescents of both sexes. This decision introduced classification bias into the study, though it was not recognized as a study limitation: children are biologically different than adolescents because they have not gone through puberty, and there are important and significant differences between the sexes concerning the cardiovascular system [8].

Boys had higher pooled prevalence than girls [9,10]. There are possible explanations for differences between the sexes: 1) the boys had a higher accumulation of visceral fat and intra-abdominal fat than girls [11], and visceral fat has been associated with higher sympathetic activity [12,13] This activation is a key mechanism underlying the effect of intra-abdominal fat accumulation on the development of hypertension [14]. For example, increased sympathetic flow may increase sodium re-absorption and subsequent increased peripheral vascular resistance resulting in increased blood pressure [14]. Also, this increased sympathetic activation can be caused by increased testosterone concentrations in males. Testosterone, acting as a mediator of the androgen receptor gene function [15], has been associated not only with increased visceral fat but also with greater vasomotor sympathetic tone and blood pressure in adolescent boys, compared to girls [16]. Therefore, we believe that the cutoffs used are not appropriate for the above and highlight the need for the scientific community to develop better diagnostic criteria and methodological quality appropriate for each sex and age of this important indicator of the cardiovascular system.

According to the title of the article, the authors’ objective was to analyze the impact of RHR for screening metabolic dysfunctions and also to identify its significance in adolescents.. For this, they used two different statistical models in order to analyze the same set of variables, one for continuous data, and another for categorical data. We found this odd, since assumptions for statistical models are quite distinct (binary logistic regression model vs. linear regression model). So we raise the following questions: *"Were the linear models used because no association was found with categorical variables? Why were the two models used? Why analyze variables with continuous data and then analyze these variables with categorical data, sequentially?"* We performed these questions, because according the objectives; the authors wanted determine the correlation between RHR and metabolic dysfunctions and also the potential power of screening the RHR. What is not clear is the use of logistic regression to meet those aims. In some instances we recommended that the authors state why they have used these tests and provide a reference for a definitive description for readers [17].

With regard to OR estimates using binary logistic regression, the literature shows that the use of OR (estimated with logistic regression) as a measure of effect in the cross-sectional studies has limitations: OR overestimates RP/RR according to increases of prevalence/incidence of outcome; between 5% and 10% OR has good approximation with RP/RR, after that the risk value is very distorted and it serves more to show the association direction (risk or protection) and not its magnitude; this topic was widely discussed in the nineties by experts [18-20], and confirms that OR overestimates the magnitude of the associations between exposures and outcomes, particularly in high prevalence [21,22]. The mathematical model for logistic regression was developed in the 1970s and 1980s to analyze case–control studies and used as a *proxy* for relative risk [23,24], where it is not possible to estimate prevalence, another important methodological factor neglected by the authors.

The authors say they used a sampling process involving two random stages (schools in the first stage and individual classes in the second stage), but give no further details of this process, for example, whether the complex sample has good accuracy. When using complex samples the design effect (*deff*) helps to estimate how accurate the sample was [25-27]. When the sampling process is not accurate the analyses need to be adjusted for the complexity of the sample, and the lack of this setting also impacts the associations [28]. Therefore, the impact of risk factors estimated by the logistic models, even without statistical significance, may not be exactly the absence shown by adjusting the primary sampling unit.

We found the use of RHR to screen for alterations in glucose and triglycerides interesting but, according to the data presented, we believe that there is no evidence for this. Accuracy (AUC) for high glucose was 0.611 (95% CI 0.534–0.688) and high triglycerides, 0.618 (95% CI 0.531–0.705), both with p-values < 0.05, but with low discrimination power—note the lower confidence bound in some cases is very close to 0.50 (random event). In other words, if we consider random variations within the CI bounds of AUC, determining the presence or absence of high glucose and high triglycerides will be as precise as playing a game of heads or tails. With regard to the accuracy of results, Swets [29] suggested operational cut-off points: the test can be non-informative/test equal to chance (0.5AUC < 0.7); moderately accurate (0.7 > AUC ≤ 0.9); highly accurate (0.9 > AUC < 1.0); and perfect discriminatory tests (AUC = 1.0).

Nowadays a "p-value < 0.05" or significant association is commonly employed to illustrate the importance of latest scientific finding. We emphasize, however, that statistical significance is neither a necessary nor a sufficient condition for proving a scientific result [30]. P-values are often used to emphasize the certainty of data, but they are only a passive read-out of a statistical test and do not take into account how well an experiment was designed, for example [31]. Goodman [32], in his "The P Value Fallacy" explains about the apparent inconsistency in much medical research, where by studies are designed according to a Neyman-Pearson statistical approach (eg. based on formal decision making and long-run evaluation of the inferential procedures), fixing statistical parameters as significance level and power, but are then analyzed by using a Fisherian point of view (eg. computing p-values and making inference based on its value, in comparison to common thresholds).

We must remember that the screening is conceptually defined as tests performed on apparently healthy people to identify those at an increased risk of a disease or disorder [33]. According to the literature, for screening to be accurate, a good screening test must have high sensitivity (few false-negative results) and a high specificity (few false-positive results) [34] and even very good tests have poor positive predictive value when applied to low-prevalence populations [35].

We would like to emphasize that Fernandes et al. [1] have provided an important scientific contribution with their study on RHR, and that criticism is an integral part of scientific progress. As the pediatrician John Locke said, "…every step the mind takes in its progress towards knowledge makes some discovery, which is not only new, but the best too, for the time at least".

## Summary

The main indicators that must be taken into account for evaluation of a screening test to measure the potential for discrimination for a common variable (population with outcome vs. no outcome population) are: sensitivity, specificity, accuracy, positive predictive value and negative predictive value. The measures of argumentation equality (CI) or difference (p-valor) are important to validate these indicators but do not indicate quality of screening.

We believe the statistical methodologies employed in support of science should consider the objectives of the paper, type of data available (with the least possible transformations) and statistical assumptions in order to answer scientific hypotheses. The interpretation of statistical data has to be made very carefully, otherwise science loses its footing and becomes a relentless pursuit of the "p-value < 0.05".

## Abbreviations

RHR: Resting heart rate; HR: Heart rate; p-value: Descriptive level; deff: Design effect; CI: Confidence interval; AUC: Accuracy.

## Competing interests

The remaining authors state no competing interest.

## Authors’ contributions

ACFM and AJFC made substantial contributions to the conception and interpretation of the material; ACFM, HBC, AJFC, LAM were involved in drafting the manuscript and revising it critically for important intellectual content and approval of the version to be published.

## Pre-publication history

The pre-publication history for this paper can be accessed here:

http://www.biomedcentral.com/1471-2431/14/117/prepub
